# Missouri Valley

**Published:** 1898-05

**Authors:** W. A. Heck

**Affiliations:** Secretary


Secretary.

### MISSOURI VALLEY VETERINARY MEDICAL ASSOCIATION.

The fifteenth regular meeting was held in the lecture-hall of the Kansas City Veterinary College on the evening of February 9, 1898. The meeting was called to order by First Vice-President Dr. R. C. Moore. Members present: Drs. S. Stewart, B. F. Kaupp, R. P. Steddom, G. A. Johnson, G. C. Pritchard, E. H. Biart, J. B. Black, R. C. Moore, F. W. Hopkins, J. H. Cock, and W. A. Heck. Visiting members of the profession were Drs. W. R. Cooper, of Kansas City, S. T. Miller, of Shelby, Iowa, and about twenty of the college students.

The first business to come before the Association was relative to members in arrears. Dr. Pritchard moved that all members who have been active members and who have moved too far away to attend the meetings, and have paid up their dues reasonably close, be elected honorary members; seconded and carried. Dr. Stewart moved that all members who are in arrears two years, and who have never paid anything to the Society, be suspended; seconded and carried. It was moved that all members suspended for non-payment of dues may be reinstated on payment of dues to date of suspension; seconded and carried.

It was moved that a committee of three be appointed to draft resolutions to be sent to the U. S. Senators from Kansas and Missouri, urging the defeat of the antivivisection bill now pending before the Senate. Motion seconded and carried, and the following committee appointed: Drs. S. Stewart, G. C. Pritchard, and G. A. Johnson.

The first paper was by Dr. Pritchard, on "Mechanical Treatment of Lameness." The doctor was of the opinion, ten or twelve years ago, that Robert Bonner's theories of a balanced foot were wild and visionary; but practical experience had forced him to accept them, and is of the opinion that any veterinarian who will give study and thought to the matter in an unbiased manner will be convinced. After giving his theories on cause of spavin, wind-puffs, and navicular disease, and the treatment of same by paring the feet, the paper was open for discussion. Dr. Johnson promptly took issue with the essayist, and a lively discussion followed for nearly an hour. Other members offered suggestions and asked questions until the subject was thoroughly turned over and looked at from many points of view. It was evident that Dr. Pritchard had given this particular subject more earnest study than any other member present.

The next paper, by Dr. E. H. Biart, on "Torsion or Displacement of the Large Colon in Protracted Colic," was very well received. It seemed to be a new idea to most of the veterinarians; consequently but few could discuss it. The doctor was deluged with questions, which he answered very satisfactorily. His method of diagnosis is manual examination per rectum, when, by feeling for the longitudinal bands on the colon, he is able to determine whether there is torsion or a normal position. If normal the band runs parallel with the abdomen. His method of replacement is by simply grasping the organ and by traction and plenty of patience, bodily turn the organ. He gave as his opinion that a large percentage of fatal cases of colic is due to this cause. He had held in the past few months post-mortems on nineteen cases, and found torsion in six of them.

Dr. R. C. Moore gave an interesting talk on the methods of one —— Giles, of this city, who is pushing a proprietary remedy with which he claims to cure all the diseases horseflesh is heir to, and interfering seriously with regular veterinary practice. He calls upon the owner of every sick animal of which he can learn, and urges the merits of his lotion, and insists upon his giving it a trial. An analysis of this remedy revealed its composition to be camphor, five grains; sulphuric ether, one and one-eighth drachms, and linseed oil enough to make one ounce. The doctor had prepared some of the lotion and had it on exhibition, and it was impossible to distinguish between this preparation and the original "Giles Lotion." Various means of dealing with this gentleman were discussed, and Dr. Moore concluded to furnish his customers at cost all of this preparation they might wish; and it is his opinion that a bottle such as is sold for one dollar he could compound for a few cents.

Dr. Stewart gave an account of a peculiar disease he calls "Contagious Vulvitis of Cattle." A large herd of heifers originally from near Trinidad, Colorado, was shipped to Kansas City, and sold in small bunches to farmers in various sections, where it seems they all developed the disease, which ran a very rapid course, and in case it was not treated terminated in death in about three weeks. None of the members had seen anything similar, but Dr. Pritchard had seen an affection of the vulva caused by feeding on old straw.

Several members had cases they wished to report, but the lateness of the hour prevented, and a motion to adjourn ended another very profitable meeting.

W. A. HECK,
Secretary.

